# Intraperitoneal Rupture of Ectopic Varices—a Rare Complication of Portal Hypertension

**DOI:** 10.1155/1994/82537

**Published:** 1994

**Authors:** Alastair N. J. Graham, Paul Mcaleese, R. John Moorehead

**Affiliations:** 1 Department of Surgery Ards Hospital Church Street Newtownards County Down Ireland; 2 Surgical Registrar Belfast City Hospital Belfast BT9 7AB Ireland

## Abstract

A 50 year old man presented with sudden abdominal pain, abdominal distension and shock. At emergency laparotomy a large amount of blood was found in the peritoneal cavity. There was micronodular cirrhosis of the liver and the spleen was enlarged. The bleeding was traced to distended veins in the right paracolic gutter which were oversewn and the abdomen closed. A coagulopathy was diagnosed and treatment including high dose aprotinin commenced. However, he continued to bleed and at a second laparotomy the area of previous haemorrhage was packed. Further deterioration continued until death 12 hours later. Intraperitoneal haemorrhage from ectopic varices is a rare occurrence. There is a high mortality rate usually due to an advanced coagulopathy. This is the first report of aprotinin being used in an attempt to treat this. On the basis of this report aprotinin would not seem to be of benefit for this condition.


*HPB Surgery*, 1994, Vol. 7, pp. 315–318
Reprints available directly from the publisher
Photocopying permitted by license only
© 1994 Harwood Academic Publishers GmbH
Printed in the United States of America

### CASE REPORT

### INTRAPERITONEAL RUPTURE OF ECTOPIC VARICES — A RARE COMPLICATION OF PORTAL HYPERTENSION

ALASTAIR N.J. GRAHAM, PAUL MCALEESE and R. JOHN MOOREHEAD

*Department of Surgery, Ards Hospital, Church Street, Newtownards, County Down, Northern Ireland*

A 50 year old man presented with sudden abdominal pain, abdominal distension and shock. At emergency laparotomy a large amount of blood was found in the peritoneal cavity. There was micronodular cirrhosis of the liver and the spleen was enlarged. The bleeding was traced to distended veins in the right paracolic gutter which were oversewn and the abdomen closed. A coagulopathy was diagnosed and treatment including high dose aprotinin commenced. However, he continued to bleed and at a second laparotomy the area of previous haemorrhage was packed. Further deterioration continued until death 12 hours later. Intraperitoneal haemorrhage from ectopic varices is a rare occurrence. There is a high mortality rate usually due to an advanced coagulopathy. This is the first report of aprotinin being used in an attempt to treat this. On the basis of this report aprotinin would not seem to be of benefit for this condition.

KEY WORDS: Portal hypertension, portasystemic shunt, peritoneum, aprotinin

### INTRODUCTION

Portal hypertension is often accompanied by dilatation of natural communications between the systemic and portal venous systems usually causing varices at the lower end of the oesophagus. Ectopic varices may form at other sites and may cause gastrointestinal haemorrhage, the source of which is often difficult to find. Bleeding into the peritoneal cavity from ectopic varices is a particularly rare feature of portal hypertension and we report such a case highlighting the difficulties in diagnosis and management of this serious condition.

### CASE HISTORY

A 50 year old caucasian male was admitted as an emergency having collapsed at home. There was a preceding history of central abdominal pain of sudden onset. He had had several previous admissions to hospital with duodenal ulceration and alcoholic hepatitis, and he drank one bottle of spirits per day.

Address correspondence to: Mr A.N.J. Graham, Surgical Registrar, Belfast City Hospital, Belfast, Northern Ireland, BT9 7AB

On examination he was conscious and was noted to be cold and diaphoretic with poor peripheral perfusion. His heart rate was 115 beats per minute, regular and blood pressure was 80/40 mm Hg. His jugulovenous pressure was not elevated and chest was clear on auscultation. There was gross abdominal distension with rigidity and absent bowel sounds. Rectal examination was normal.

Resuscitation was commenced and investigations undertaken. Haemoglobin was 2.1 gms/dl, sodium 125 mmol/l, potassium 3.0 mmol/l, urea 4.2 mmol/l, $PO_2$ 2.6 kPa, $PCO_2$ 8 kPa, pH 6.94.

A presumptive diagnosis of ruptured abdominal aortic aneurysm was made and, at emergency laparotomy carried out through a midline incision, several litres of blood were found in the abdominal cavity. The liver had the appearances of micronodular cirrhosis and the spleen was moderately enlarged but intact. After a careful search distended veins running from the retroperitoneal area to the ascending colon were identified and were bleeding at high pressure (see Figure 1). They were oversewn with absorbable suture and this controlled the haemorrhage. In view of the patient's critical condition no definitive procedure to reduce the portal pressure was possible. The abdomen was closed and the patient transferred to the intensive care department. Fluid replacement at this time totalled 12 litres of colloid, 12 units of blood and 4 units of fresh frozen plasma. Coagulation screen revealed elevated prothrombin time of 51 seconds, partial thromboplastin test greater than 240 seconds, fibrin degradation test 8 microgm/ml (normal) and platelets 277 by $10^9$/l. Aprotinin therapy was commenced with bolus injection of 1 million units and 200,000 units per hour thereafter.

After initial haemodynamic stability, his condition deteriorated with respiratory compromise secondary to splinting of the diaphragm by abdominal distension. Further transfusion of 10 units blood, 4 units fresh frozen plasma and platelets from 6 donors was given. At 10 hours post-operation he underwent a second laparotomy and the findings were of a gross amount of blood in the abdominal cavity. There was a generalized ooze of blood from around the right paracolic gutter. This was packed with large abdominal wipes and he was returned to the intensive care department. Although his condition initially improved, he underwent a further deterioration and died 12 hours after the second laparotomy. An autopsy was not obtained.

### DISCUSSION

In portal hypertension ectopic varices are most commonly reported in the duodenum, small intestine, colon, rectum and at ileostomy and colostomy sites. Intraperitoneal varices are a much rarer occurrence and we have found only 18 previously reported cases causing haematoperitoneum[1–3]. All patients had alcoholic cirrhosis and presented with sudden abdominal pain, abdominal distension and shock. However, it has been emphasized that hepatocellular carcinoma is a much commoner cause of intraperitoneal bleeding in patients with cirrhosis[1].

Because of the catastrophic nature of bleeding from intraperitoneal varices, the diagnosis is usually made at emergency laparotomy or post-mortem examination. However, duplex ultrasonography has been used to diagnose non-bleeding intraperitoneal varices in a patient with cirrhosis[4].

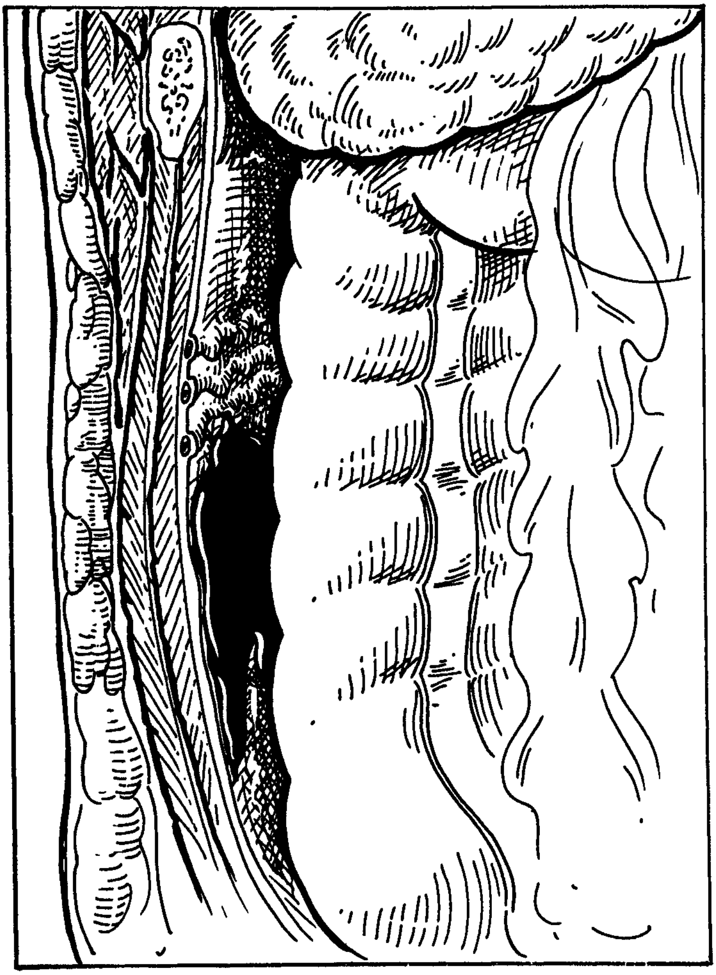

**Figure 1** Distended veins in the right paracolic gutter bleeding at high pressure.

In those patients who underwent surgery the most common procedure was ligation of the bleeding varix and this was followed by death in 9 out of 14 cases. Two reported cases have had portocaval shunts performed[5,6], however, neither of these patients survived. In our case we intended to perform a shunt if the patient's condition stabilized and the coagulopathy resolved. Unfortunately the opportunity did not arise.

Selective angiography has been reported but in most cases was unhelpful and because of the delay involved is no longer advocated[7]. Intravenous pitressin therapy has been proposed with the proviso that it does not delay surgery[7].

High doses of aprotinin have been found to significantly reduce the blood loss resulting from repeat cardiac surgery[8]. As aprotinin's efficacy is believed to be due to its antifibrinolytic activity, and our patient had no evidence of fibrinolysis, there would be no logical expectation that it would reduce bleeding in this context. However, it has been reported to reduce the blood loss in aortoiliac surgery where fibrinolysis would not be expected[9] and so there is optimism for its use in other situations involving severe haemorrhage. The current report is the first to describe its use in ruptured intraperitoneal varices and it did not seem to have a beneficial effect. It would, however, be difficult to judge the effect of a single factor given the number of different features that would determine the outcome of a case such as this.

This case illustrates the diagnostic and therapeutic difficulties in the management of bleeding intraperitoneal varices. The diagnosis should be considered in all patients with cirrhosis developing sudden abdominal pain. However, it is likely that due to the poor state of the patients at presentation, there will continue to be a high mortality rate associated with this condition.

### *Acknowledgements*

We would like to thank Brendan Ellis of the Department of Medical Illustration, Royal Victoria Hospital, Belfast for drawing the illustration.

### *References*

1. Lebrec, D. and Benhamou, J-P. (1985) Ectopic varices in portal hypertension. *Clinics in Gastroenterology*, **14**, 105–121
2. Sullivan, E.D. and Breiter, J.S. (1983) Intraperitoneal variceal bleeding: an unusual cause for hematoperitoneum. *Connecticut Medicine*, **47**, 12–14
3. Ragupathi, K., Bloom, A. and Pai, N. (1985) Hemoperitoneum from ruptured omental varices. *Journal of Clinical Gastroenterology*, **7**, 537–538
4. Jacoby, J.H. and D'Auria, D. (1991) Nodular intraperitoneal masses in a patient with cirrhosis. *New Jersey Medicine*, **88**, 46–47
5. Fawaz, K.A., Kellum, J.M. and Deterling, R.A. (1982) Intraabdominal variceal bleeding. *American Journal of Gastroenterology*, **77**, 578–579
6. Miller, J. and Dineen, J. (1968) Ruptured abdominal varix. *New England Journal of Medicine*, **278**, 508
7. Fox, L., Crane, S.A., Bidari, C. and Jones, A. (1982) Intra-abdominal haemorrhage from ruptured varices. *Arch. Surg.*, **117**, 953–956
8. Royston, D., Bidstrup, B., Taylor, K.M. and Sapsford, R.N. (1987) Effect of aprotinin on need for blood transfusion after repeat open heart surgery. *Lancet*, **2**, 1289–1291
9. Thompson, J.F., Roath, O.S., Francis, J.L., Webster, J.H.H. and Chant, A.D.B. (1990) Aprotinin in peripheral vascular surgery. *Lancet*, **335**, 911

